# Selective enrichment of heterotrophic nitrifiers *Alcaligenaceae* and *Alcanivorax* spp. from industrial wastewaters

**DOI:** 10.3934/microbiol.2020002

**Published:** 2020-02-13

**Authors:** Mārtiņš Kalniņš, Andrejs Bērziņš, Dita Gudrā, Kaspars Megnis, Dāvids Fridmanis, Pavel Danilko, Olga Muter

**Affiliations:** 1 Institute of Microbiology & Biotechnology, University of Latvia, 1 Jelgavas Str., Riga LV-1004, Latvia; 2 Latvian Biomedical Research and Study Centre, 1 Ratsupites Str, Riga LV-1067, Latvia; 3 JSC Olaine chemical plant BIOLAR, 3 Rupnicu Str., Olaine, LV-2114, Latvia

**Keywords:** activated sludge, industrial wastewaters, Ion Torrent PGM sequencing, dehydrogenase, nitrification

## Abstract

Removal of nitrogen from wastewaters (WW) represents a global problem. The low nitrification rate during WW treatment is often caused by ecotoxicity. This problem is attributed mostly to the industrial WW. Our study was focused on the testing of industrial WW and activated sludge (AS) with the aim to reveal the abundance of nitrifiers and increase their biomass, thus, providing the additional step, i.e., bioaugmentation, within the technological process of WW treatment. Plating of AS on the selective solidified media designated for the 1^st^ and 2^nd^ nitrification stages, resulted in the shift in bacterial community structure with dominated *Alcaligenaceae* and *Alcanivorax* for the 1^st^ stage, and *Alcanivorax*-for the 2^nd^ stage of nitrification, respectively. Incubation of AS in the presence of real WW and selective nitrification broth resulted in a considerable increase (one or two magnitudes in the presence of the 1^st^ and 2^nd^ stage nitrification broth, respectively) of culturable nitrifiers after 5 days incubation under aerated conditions. The obtained data provide with evidence about a possibility to strengthen the role of heterotrophic nitrifiers in the treatment of industrial WW, where toxicity obstacles inhibited nitrification under conventional conditions.

## 1. Introduction

Nitrification processes in soil and aquatic environments play an important role in N-cycle, and is strongly dependent on various factors, e.g., soil matrix, water status, aeration, temperature, pH, microbial community structure and others [1],[2]. Nitrification in recirculation aquaculture systems reduces concentrations of NH_4_^+^ and NH_3_, which are critical since these are highly toxic for fish [3]. Optimization of the treatment of agro-based industrial effluents also needs to focus on the nitrification rate, which in turn, allows reducing a hydraulic retention time [4]. Furthermore, poor nitrification in municipal wastewater (WW) treatment plants represents a serious problem leading to inefficient WW treatment [5].

Nitrification mainly involved two phylogenetically unrelated groups of autotrophic bacteria, i.e., ammonia-oxidizing bacteria (AOB) and nitrite-oxidizing bacteria (NOB). Besides, heterotrophic nitrifying and oxygen tolerant denitrifying bacteria may substantially contribute to the convertion of nitrogen [6],[7].

Meta-analysis of literature data showed that nitrification is a log-linear function of N mineralization, increasing rapidly at low mineralization rates but changing only slightly at high mineralization rates [8]. Different feed C/N ratios in wastewater treatment processes may substantially change the efficiency of nitrification and denitrification. Thus, application of feed C/N ratios of 2.7, 4.2 and 5.6 in a continuous-flow microaerobic moving bed biofilm bioreactor showed C/N ratio of 4.2 to be the most efficient [9].

Heavy metals are known to influence the nitrification process in conventional AS systems, while inhibition effects are dependent on the metal oxidation state and other physicochemical conditions [10]–[13].

One of the powerfull tools for maintaining the nitrification activity in soils and aquatic environments is the bioaugmentation with nitrifiers [14]–[16]. A great diversity of microorganisms takes part in the N-cycle, e.g., denitrifying fungi, nitrifying archaea, anammox bacteria, aerobic denitrifying bacteria and heterotrophic nitrifying microorganisms [17].

In the study with conventional AS, the bioaugmentation of AS with nitrifying AS obtained from a pilot plant performing full nitritation under stable conditions (6 % dw) resulted in significantly faster achieving the partial nitrification, compared with the non-bioaugmented AS [18]. Mannucci et al. [19] reported that bioaugmentation with nitrifiers of membrane bioreactors was efficient, while depended on the temperature and other operating conditions in seeding and seeded reactors. Nitrification bioaugmentation with sidestream granules with *Nitrosomonas* as a dominant genus, instead of flocculent biomass was suggested by [20]. Sludge aerobic granulation resulted in AOB enrichment and the reduction of nitrite-oxidizing bacteria (NOB) in granules, which is highly favorable to a stable partial nitrification operation [21].

However, bioaugmentation strongly influences the microbial community, that might affect the nitrification kinetics, due to the unequal and asynchronous increase of the ammonia uptake rate [22]. The possible side effects of bioaugmentation should be strongly controlled by optimization of the amount and physiological activity of the microbial biomass seeded, as well as technological scheme, which determines the stages and frequency of bioaugmentation.

For further development of this approach, a search for new efficient consortia of nitrifiers derived from the local site and, hence, competitive in the certain microbial communities, would be highly acknowledged. In our study, it was hypothesized that the enrichment of AS in the selective broth could reveal the abundance of nitrifiers and increase their biomass, thus, providing the additional step within the technological process, e.g., WW treatment, soil remediation and other environmental bioprocesses.

The aim of this study was to stimulate the growth of nitrifiers in the AS from the WWTP, which is operated at chemical industry. The chemical plant produces resins, drying accelerators, plasticizers, organic synthetic products, azo initiators and others. The low nitrification rate at these WWTP is caused by the presence of toxic compounds. Selective pressure of broth and aeration conditions were expected to determine the bacterial taxa in the AS, which have a potential for stimulating the nitrification process in case of bioaugmentation.

## 2. Materials and methods

The AS and WW have been sampled at JSC BIOLARS. The WW had the following physico-chemical characteristics: chemical oxygen demand (COD) 900 ÷ 1100 mg/L; biological oxygen demand (BOD5) 350 ÷ 380 mg/L; N_tot_ 60 ÷ 80 mg/L; P_tot_ 6 ÷ 8 mg/L. The dry weight (dw) of AS samples was 2.8 %. Samples were stored at 4 °C until their use in laboratory experiments.

### 2.1. Enrichment experiment in miniaturized aeration tanks

Three glass 500 mL bottles with a connected aeration system were sterilized and filled with 130 mL filtered WW, 50 mL AS, 20 mL 10x stock solution of NITR-I or NITR-II (Table 1). In the control bottle, 20 mL deionized sterile water were added. The WW with AS and medium were continuously aerated during 10 days, afterwards, during 7 days the incubation was performed with a periodical shaking, i.e., twice daily. Incubation was performed at 23 °C.

**Table 1.** The composition of media used for enrichment and detecting of nitrifying bacteria. NITR-I–the 1^st^ stage nitrification, NITR-II–the 2^nd^ stage nitrification.

Compound	NITR-I, g/L	NITR-II, g/L	Compound	NITR-I, g/L	NITR-II, g/L
(NH_4_)_2_SO_4_	2	0	FeSO_4_ · 7H_2_O	0.4	0.03
NaNO_3_	0	0.006	CaCO_3_	5.0	1
K_2_HPO_4_	1	1	CaCl_2_	0	0.3
MgSO_4_·7H_2_O	0.5	0.1	Agar*	15	15
NaCl	2	0.3			

*Agar was added only to solidified media for CFU counting.

### 2.2. Enumeration of culturable microorganisms

For enumeration of the colony forming units (CFU) in AS, the following media were used: Standard Method Agar (StMA, BD, USA) for the total count of aerobic heterotrophic bacteria; selective media for enumeration of nitrifiers responsible for the 1^st^ and 2^nd^ stages of nitrification, i.e., NITR-I and NITR-II, respectively (Table 1). The microdilution plating method was used, i.e., decimal dilutions of samples were prepared in 0.85 % NaCl in microplates, afterwards 10-*µ*L droplets from each dilution were plated on solidified media [23] The number of CFU was determined after incubation at 30 °C for 48h (StMA) and 96h (NITR-I and NITR-II).

### 2.3. Enzyme activity of microorganisms

Dehydrogenase (DHA) assay was applied for visualization of CFUs on NITR-I and NITR-II media. The reaction mixture (40 mg INT(2-p-iodo-3-nitrophenyl-5-phenyltetrazolium), 10 mg glucose in 20 mL of 0.25M TRIS) was applied on the surface of the solidified medium with colonies [24]. The color of colonies turned purple after 20 min incubation, as a result of INT reduction to iodonitrophenylformazan [25] (Figure S1).

### 2.4. Ion torrent PGM sequencing

DNA was extracted using FastDNA SPIN Kit for Soil (MP Biomedicals, USA). Polymerase chain reaction (PCR) of 16S rRNA V3-4 region was performed employing Probio_Uni_F and Probio_Uni_R [26] primers tagged with 10-11bp unique barcode labels along with the adapter sequence. PCR reaction was carried out using Phusion Hot Start II DNA Polymerase (Thermo Fisher Scientific, USA) and GeneAmp® PCR System 9700 (Thermo Fisher Scientific, USA) according to manufacturers' guidelines. Thermal conditions of the PCR reaction were set as follows: 98 °C for 30 seconds, 35 cycles of 98 °C for 10 seconds, 67 °C for 15 seconds, 72 °C for 15 seconds with a final extension at 72 °C for 7 minutes. PCR products were purified using NucleoMag® NGS Clean-Up and Size Select Kit (Macherey-Nagel, Germany). The quality and quantity of amplicons were assessed using Agilent High Sensitivity DNA kit and Agilent 2100 BioAnalyzer (Agilent Technologies, USA). Samples were diluted to 12 pM and pooled. Samples were sequenced on Ion Torrent Personal Genome Machine sequencing platform employing Ion PGM™ Hi-Q™ View OT2 kit (Life Technologies, USA) for template generation and Ion PGM™ Hi-Q™ View Sequencing kit (Life Technologies, USA) on Ion 318 v2 chip (Life Technologies, USA). 

### 2.5. Mathematical statistics and data analysis

Sequencing data analysis was carried out using QIIME version 1.8.0 and UPARSE pipeline version 7.0.1001 to quality-filter and cluster 16S rRNA amplicon sequences [27]. Sequences with the mean sequence quality score > 20 passed the quality control. Operational Taxonomic Units (OTUs) were built at 97 % sequence identity with UCLUST algorithm [28]. Taxonomic assignment to the lowest possible rank was performed with RDP (Wang et al., 2007), using the Greengenes [29] (http://greengenes.secondgenome.com) reference dataset (gg_otus-13_8 release). Alpha diversity metric Shannon index was calculated within the QIIME environment.

## 3. Results and discussion

Preliminary testing of nitrification activity in the WW indicated to the relatively low activity of nitrifiers, particularly shown by a potential ammonium oxidation assay (data not shown). This problem could be associated with abundance of toxic compounds in WW. The primary task of this study was to reveal the microorganisms in AS with nitrification potential, which afterwards could serve for bioaugmentation of this WWTP.

### 3.1. Shift in bacterial community structure of AS after cultivation in the selective nitrification media

**Figure 1.**
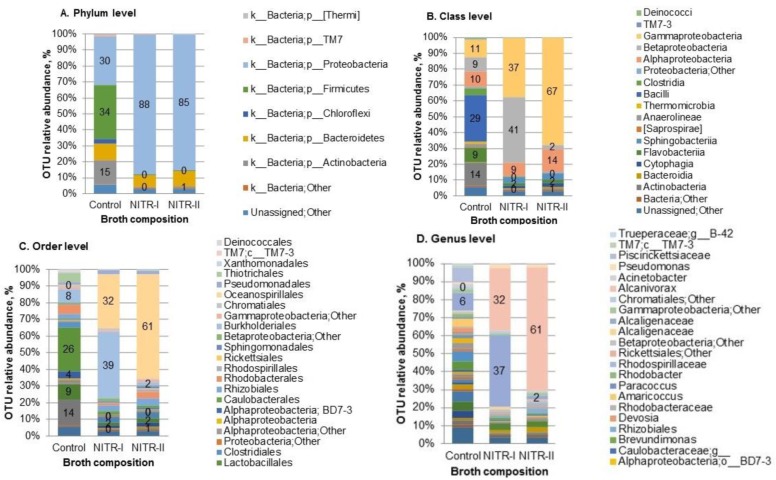
Bacterial community composition in the intact activated sludge (AS) and after cultivation on the selective solidified media. NITR-I–incubation on the medium for the 1^st^ step of nitrification; NITR-II–incubation on the medium for the 2^nd^ step of nitrification. The values <1% are not indicated in the diagram.

Composition of bacterial community in the intact AS showed a relatively high diversity, comparing with enriched samples NITR-I and NITR-II, with Shannon diversity indexes of 7.7, 5.3 and 4.5, respectively (Table S1). At the phylum level the intact AS consisted of Firmicutes (34%), Proteobacteria (30%), Actinobacteria (15%), Bacteroidetes (10%) and others, which were represented with a relative abundance below 10% (Figure 1A). These data corraborate with other studies, which reported about the dominance of Proteobacteria, Actinobacteria, Bacteroidetes, Firmicutes and Verrucomicrobia phyla at municipal WWTP [30].

Incubation of AS in NITR-I and NITR-II resulted in a considerable dominance of Proteobacteria with relative abundance of 88% and 85%, respectively (Figure 1A). After incubation in NITR-I, Proteobacteria at class level was represented mostly by Gammaproteobacteria (37%) and Betaproteobacteria (41%), while after incubation in NITR-II – by Gammaproteobacteria (67%) (Figure 1B). At order level it was shown, that Betaproteobacteria and Gammaproteobacteria were represented by Burkholderiales and Oceanospirillales, respectively (Figure 1C). In turn, at genus level–*Alcaligenaceae* (genus was not recognized) and *Alcanivorax*, respectively (Figure 1D).

Typical AOB in WW are known to be *Nitrosomonas* and *Nitrosospira* species [30],[31]. However, in the high strength industrial WW containing enhanced concentrations of ammonia as well as other toxic compounds, these typical nitrifiers do not survive [32]. On the other hand, there is a probability that the bias could be introduced during the bioinformatic analysis as the public sequence databases are still under development and contain various errors, thus inducing inaccurate results regarding to the assignment of individual OTUs [33],[34]. In this manner portion of typical AOB related sequences might be assigned to other taxonomical branch based on sequence homology as described previously (Ye et al., 2011). Another possible loss of data resolution might arise due to misassignment to OTUs, which contain only a few sequences. However, the occurrence of this type of error is low, since, during the data analysis, low abundance sequences are discarded from further processing [35].

In the study with municipal WW, nitrification rate of the AS increased with increasing O_2_, whereas denitrification occurred mainly in the anoxic zones [36]. Although the treatment of AS in the membrane reactor under low dissolved oxygen level also resulted in a sustained effective simultaneous nitrification/denitrification [37].

In turn, heterotrophic nitrification and aerobic denitrification, which occur simultaneously, have the following mechanisms: NH_4_^+^→NH_2_OH→NO_2_^−^→NO_3_^−^ and NO_3_^−^→NO_2_^−^→N_2_O→N_2_ [38],[39]. It was shown that *Acinetobacter calcoaceticus* isolated from a membrane bioreactor, converted NH_4_^+^ to N_2_ under aerobic conditions during heterotrophic growth. Nitrate reductase and nitrite reductase activity were not detectable in this reaction mixture. Authors suggested the nitrogen removal under tested conditions may occur via a hydroxylamine intermediate instead of nitrite [40].

**Figure 2.**
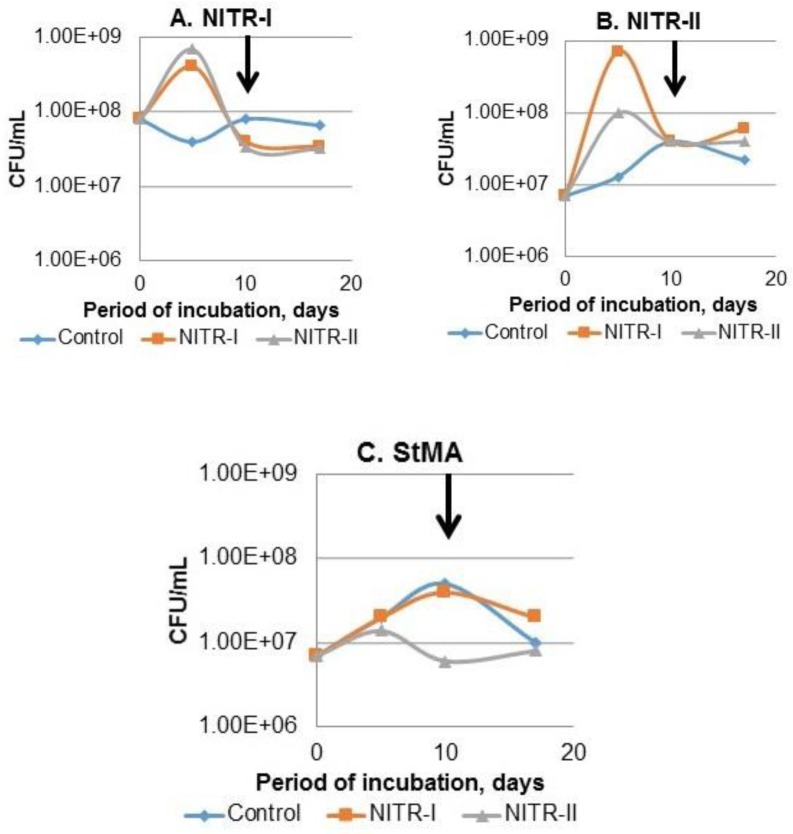
Influence of broth composition and aeration on the concentration of culturable nitrifying and other bacteria in AS. Control–AS and WW; NITR-I and NITR-II – AS and WW amended with the broth for the 1^st^ and 2^nd^ step of nitrification, respectively. StMA – medium for the enumeration of the total number of aerobic heterotrophic bacteria. The continuous aeration was turn out after the first 10 days incubation (showed with arrow). Values are the average of two measurements.

As reported by [41], among the dominant taxa responsible for the heterotrophic denitrification processes in WW were *Burkholderiadaceae*, *Comamonadaceae*, *Alcaligenaceae*. Our data indicated that the conditions for the 1^st^ nitrification stage (NITR-I) were the most preferable for *Alcaligenaceae*, where its relative abundance reached 37 %, while in the presence of NITR-II it was only 2% (Figure 2D). According to literature data, *Alcaligenaceae* are capable of nitrification and denitrification, thus, providing a possible advantage in biological WW system [6]. The presence of nitrous oxide reductase gene confirmed the presence of oxygen-tolerant denitrification system in these bacteria. Heterotrophic nitrification of ammonia by *Alcaligenes* sp. into nitrite is supposed to occur via formation of hydroxyl amine, which is oxidized to nitrous oxide using oxygen or nitrite as electron acceptor [6]. *Alcaligenes faecalis* was shown to be efficient heterotrophic nitrifier and aerobic denitrifier in a high-strength ammonium WW, where 40% and 60% of ammonium were converted to N_2_ gas and cell mass, respectively [42]. Excess biomass of *Alcaligenaceae* produced during WW treatment process was suggested to apply in agriculture. *Alcaligenes faecalis* showed antagonistic behaviour to plant pathogens [43].

Another important taxa, i.e., *Alcanivorax* spp., in our study was notably developed under selective pressure of NITR-I and NITR-II, compared to the control, i.e., 32%, 61% and <1%, respectively (Figure 2D). As was reported in literature, these bacterial species are known to reduce nitrate to nitrite. For example, *Alcanivorax* has been isolated from the deep-sea sediment and characterized as halophilic, aerobic, Gram-negative, non-spore-forming, catalase- and oxidase-positive motile rods, growing on a restricted spectrum of organic compounds, including some organic acids and alkanes [44]. Alcanivorax bear the alkane monooxygenase (alkB) gene encoding the Alk enzyme, and, hence, attract a great attention in terms of treatment for oil-polluted WW [45]. Nakano et al. [46] reported that *Alcanivorax* spp. was isolated from organically enriched marine sediments, and dominated in the bacterial consortia responsible for nitrogen removal.

### 3.2. Concentration of culturable bacteria in AS during incubation in real WW amended with nitrification broth

Incubation of AS in the liquid phase with different composition was expected to change the amount of culturable microorganisms in AS. The data on CFU counts for nitrifiers of the 1^st^ and 2^nd^ stages, as well as “total” aerobic heterotrophic bacteria are presented in Fig.2. The number of CFU obtained on StMA showed, that aeration stimulated the proliferation of the heterotrophs in the control and NITR-I bottles during the first 10 days from 7.0*10^6^ up to 5.1*10^7^ and 4.0*10^7^ CFU/mL, respectively (Figure 2C). Interestinlgy, the CFU counts on the selective media (NITR-I and NITR-II) was found to be higher than on StMA, which reached 10^9^ magnitude in the bottles amended with NITR-I and NITR-II broth after 5 days incubation. In particular, this maximum of CFU count was obtained in the both, i.e., NITR-I and NITR-II bottles for the 1^st^ stage nitrifiers, while for the 2^nd^ stage nitrifiers surprisingly the bottle NITR-I was found as the more favourable among three types of liquid media (Figure 2B). The initial pH values in the control, NITR-I and NITR-II bottles were 4.71; 7.61 and 8.53, respectively. After 17 days incubation, the pH level gradually increased to 8.30; 8.42 and 9.11, respectively.

The data obtained in this experiment, indicated that complex interrelations between physicochemical characteristics of real WW, composition of nutrient amendments, as well as physiological activity of nitrifiers and other microorganisms (derived from AS), resulted in dynamic changes upon incubation. The mechanisms of these changes are hardly understood and needs to be studied in further experiments.

## 4. Conclusions

Plating of AS on the selective solidified media designated for the 1^st^ and 2^nd^ nitrification stages, resulted in the shift in bacterial community structure with dominated *Alcaligenaceae* and *Alcanivorax* for the 1^st^ stage, and *Alcanivorax* - for the 2^nd^ stage of nitrification, respectively. These Proteobacteria are known to be heterotrophic nitrifiers and could be used for stimulation of nitrogen removal from WW by means of bioaugmentation.

Incubation of AS in the presence of real WW and selective nitrification broth resulted in a considerable increase (one and two magnitudes in the presence of NITR-I and NITR-II, respectively) of culturable nitrifiers after 5 days incubation under aerated conditions. The obtained data provide with evidence about a possibility to strengthen the role of heterotrophic nitrifiers in the treatment of industrial WW, where toxicity obstacles inhibited nitrification under conventional conditions.
