# Supplementary material for: Selective enrichment of heterotrophic nitrifiers Alcaligenaceae and Alcanivorax spp. from industrial wastewaters
Source: AIMS Microbiol. 2020 Feb 13;6(1):32–42. doi: 10.3934/microbiol.2020002 (PMC7099200; doi:10.3934/microbiol.2020002)
Supplement: Supplementary file 1 [file microbiol-06-01-002-s001.pdf]

AIMS Microbiology

AIMS Microbiology, 6(1):32–42.
DOI: 10.3934/microbiol.2020002
Received: 31 December, 2019
Accepted: 06 February, 2020
Published: 13 February, 2020

http://www.aimspress.com/journal/microbiology

Research article

Selective enrichment of heterotrophic nitrifiers *Alcaligenaceae* and *Alcanivorax* spp. from industrial wastewaters

Mārtiņš Kalniņš[1], Andrejs Bērziņš[1], Dita Gudrā[2], Kaspars Megnis[2], Dāvids Fridmanis[2], Pavel Danilko [3] and Olga Muter[1*]

[1] Institute of Microbiology & Biotechnology, University of Latvia, 1 Jelgavas Str., Riga LV-1004, Latvia
[2] Latvian Biomedical Research and Study Centre, 1 Ratsupites Str, Riga LV-1067, Latvia
[3] JSC Olaine chemical plant BIOLAR, 3 Rupnicu Str., Olaine, LV-2114, Latvia

* **Correspondence:** olga.muter@inbox.lv; Tel: +37126846647; Fax: +37167033924.

Abstract: Removal of nitrogen from wastewaters (WW) represents a global problem. The low nitrification rate during WW treatment is often caused by ecotoxicity. This problem is attributed mostly to the industrial WW. Our study was focused on the testing of industrial WW and activated sludge (AS) with the aim to reveal the abundance of nitrifiers and increase their biomass, thus, providing the additional step, i.e., bioaugmentation, within the technological process of WW treatment. Plating of AS on the selective solidified media designated for the 1st and 2nd nitrification stages, resulted in the shift in bacterial community structure with dominated *Alcaligenaceae* and *Alcanivorax* for the 1st stage, and *Alcanivorax*-for the 2nd stage of nitrification, respectively. Incubation of AS in the presence of real WW and selective nitrification broth resulted in a considerable increase (one or two magnitudes in the presence of the 1st and 2nd stage nitrification broth, respectively) of culturable nitrifiers after 5 days incubation under aerated conditions. The obtained data provide with evidence about a possibility to strengthen the role of heterotrophic nitrifiers in the treatment of industrial WW, where toxicity obstacles inhibited nitrification under conventional conditions.

Keywords: activated sludge; industrial wastewaters; Ion Torrent PGM sequencing; dehydrogenase; nitrification

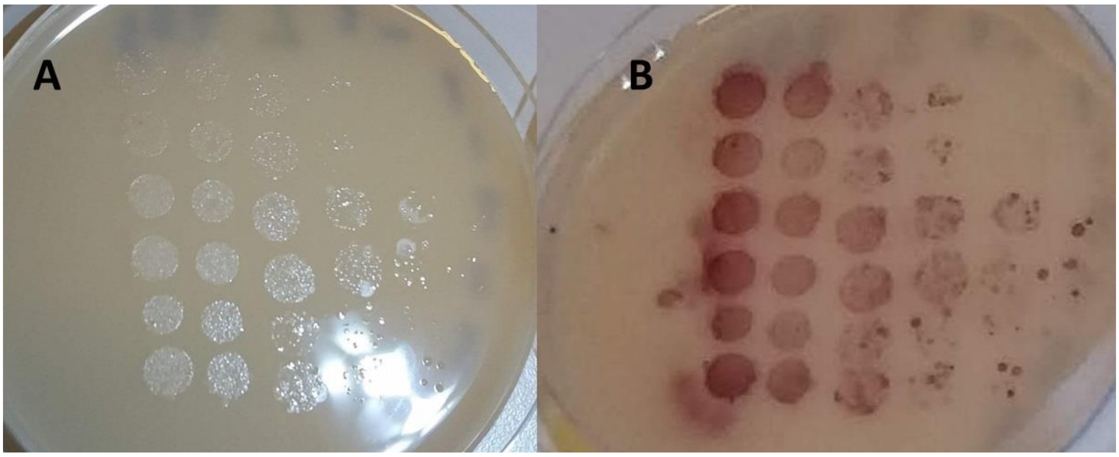

Figure S1. Colonies of nitrifiers formed on the solidified medium for nitrification phase I. A, colonies after 4 days of incubation; B, colonies after 30 min reaction with the reaction mixture for DHA assay [24].

Table S1. Qualitative information on samples sequenced by Ion Torrent PGM. Sample abbreviation, number of sequences obtained after sequencing, after quality filtering and number of OTUs found per each sample with the respective Shannon index is shown.

Sample	Number of sequences obtained	Number of sequences retained after quality control	Number of OTUs	Shannon index
Control	420506	78887	2989	7.72
NITR-I	307773	67727	1181	5.27
NITR-II	523781	119412	1828	4.97

Supplement M1. Ion Torrent PGM sequencing

DNA extraction. DNA was extracted from a total of seven samples using FastDNA SPIN Kit for Soil (MP Biomedicals, USA). The samples were first centrifuged at 4000 rpm for 10 minutes at +4ºC, and the supernatant was discarded. The following protocol was performed according to the manufacturer's guidelines. The concentration of extracted DNA was measured using Qubit® 2.0 Fluorometer High Sensitivity Assay (Life Technologies, USA), while the amount, the average size and quality of the DNA were assessed using electrophoresis on 1.2% agarose gels.

Polymerase chain reaction. Primers were designed for the PCR amplification of 16S rRNA V3 region specific to the domain bacteria. DNA was amplified separately by reverse (Probio_Uni_R 5'-ATTACCGCGGCTGCT-3') and forward (Probio_Uni_F 5'-CCTACGGGRSGCAGCAG-3') primers as previously described. Both primers were tagged with 10-11 bp unique barcode labels along with the adapter sequence (5'-CCATCTCATCCCTGCGTGTCTCCGAC-3') to allow multiple samples to be included in a single sequencing run.

PCR amplification was carried out using a GeneAmp® PCR System 9700 (Thermo Fisher Scientific, USA). The PCR conditions were the following: 98°C for 30 seconds, 35 cycles of 98°C for

10 seconds, 67°C for 15 seconds, 72°C for 15 seconds with a final extension at 72°C for 7 minutes. 16S rRNA PCR products were then quantified, pooled, and purified for the sequencing reaction using NucleoMag® NGS Clean-Up and Size Select kit (Macherey-Nagel, Germany). The quality and acquired amount of 16S rRNA V3 amplicons were assessed using Agilent DNA High Sensitivity DNA kit on Agilent 2100 BioAnalyzer (Agilent Technologies, USA).

16S sequencing analysis. Prior to clonal amplification, each library was subsequently diluted to 12 pM and pooled. The sample emulsion PCR, emulsion breaking, and enrichment were performed using the Ion PGM™ Hi-Q™ OT2 Kit (Life Technologies, USA) following the manufacturer's instructions. The input concentration of the DNA template copy/Ion Sphere Particles (ISPs) was added to the emulsion PCR master mix, and the emulsion was generated using the One Touch DL (Life Technologies, USA). Next, ISPs were recovered and template-positive ISPs enriched using Dynabeads MyOne™ Streptavidin C1 beads (Life Technologies, USA). The ISP enrichment was confirmed using a Qubit 2.0 fluorometer (Life Technologies, USA). The complete sample was loaded onto 318 v2 chip and sequenced on the PGM for 850 cycles employing the Ion PGM™ Hi-Q™ Sequencing Kit to achieve 400bp reads. Bidirectional sequencing was performed (i.e. sequence reads started from forward and reverse PCR primers), but reads were not paired. Each run was expected to produce approximately 300,000 reads. After the sequencing run was completed, the individual sequence reads were filtered by the PGM software to remove low quality sequences. Sequences matching the PGM 3' adaptor were automatically trimmed. All PGM quality-approved, trimmed, and filtered data were exported as bam files.

Data analysis. The analysis of sequencing data was carried out using QIIME v.1.8.0. and UPARSE v.7.0.1001. pipeline to quality-filter and cluster 16S rRNA amplicon sequences (Pylro et al., 2014). The sequences with the mean sequence quality score >20 were accepted according to quality control criteria. Operational Taxonomic Units (OTUs) were built at 97% sequence identity with uclust (Edgar, 2010). Taxonomic assignment to the lowest possible rank was performed with RDP, using the Greengenes (http://greengenes.secondgenome.com) reference dataset (gg_otus-13_8 release). Alpha diversity metric Shannon index (e.g. within sample diversity) was calculated within the QIIME environment.

AIMS Press

© 2020 the Author(s), licensee AIMS Press. This is an open access article distributed under the terms of the Creative Commons Attribution License (http://creativecommons.org/licenses/by/4.0)
